# Diversity and prevalence of ticks associated to cattle and wild animals in lagoon ecosystems of northern Veracruz, México

**DOI:** 10.1007/s10493-026-01145-8

**Published:** 2026-05-27

**Authors:** Anali Elsa Bello-Velázquez, Miguel Ángel Alonso-Díaz, Dora Romero-Salas, Gerardo Suzán, Juan Manuel Pech Canché, Carmen Guzmán-Cornejo

**Affiliations:** 1 Facultad de Medicina Veterinaria y Zootecnia, Universidad Nacional Autónoma de México, Ciudad de Mexico, Mexico; 2 Facultad de Ciencias Biológicas y Agropecuarias, Universidad Veracruzana, Carretera Tuxpan - Tampico, km 7.5, Tuxpan, Veracruz, Mexico; 3 Facultad de Medicina Veterinaria y Zootecnia, Centro de Enseñanza, Investigación y Extensión en Ganadería Tropical, Universidad Nacional Autónoma de México, Km. 5.5 Carretera Federal Tlapacoyan-Martínez de La Torre, Martínez de La Torre, 93600 México; 4 Facultad de Medicina Veterinaria y Zootecnia, Laboratorio de Parasitología Veterinaria, Universidad Veracruzana, Veracruz, Mexico; 5 Facultad de Medicina Veterinaria y Zootecnia, Departamento de Etología, Fauna Silvestre y Animales de Laboratorio, Unidad Mérida, Universidad Nacional Autónoma de México, Ciudad de Mexico, Mexico; 6 Facultad de Ciencias Biológicas y Agropecuarias, Laboratorio de Vertebrados Terrestres, Universidad Veracruzana, carret. Tuxpan-Tampico, km 7.5, Tuxpan Veracruz, Mexico; 7 Facultad de Ciencias, Laboratorio de Acarología, Universidad Nacional Autónoma de México, Circuito Exterior s/n, Ciudad de México, 04510 México

**Keywords:** Wildlife, Lxodida, Hard ticks, Parasitism

## Abstract

Understanding the diversity of ticks is crucial to comprehending their population dynamics and role in pathogen transmission. Between July 2022 and July 2023, a total of 440 animals were inspected across 35 Cattle Production Units (CPU) adjacent to the Tamiahua and Tampamachoco lagoons in northern Veracruz, Mexico. A comprehensive total of 7,026 ticks, encompassing various developmental stages, were collected; this total includes 5,854 adults (83.3%), 984 nymphs (14%), and 188 larvae (2.7%). The prevalence of cattle infested with ticks was 93.7%, while in wild animals it was 50.9%. Morphological identification revealed the presence of eight species from three genera: *Rhipicephalus microplus*, *Haemaphysalis leporispalustris*, and six species of the genus *Amblyomma* (*A*. *auricularium*,* A*. *dissimile*, *A*. cf. *tenellum*, *A*. *mixtum*, *A*. *ovale*, and *A*. *rotundatum*). In wild animals, greater species richness was recorded with *A*. *auricularium* being the dominant species. In contrast, only two species were found in cattle. Interaction network analysis showed that *A*. *mixtum* has the highest number of associations with different hosts such as *Bos taurus*, *Didelphis virginiana*, *Dasypus novemcinctus*, *Procyon lotor* and *Sylvilagus floridanus*, while *R*. *microplus* was associated with *Bos taurus* and *D*. *virginiana*. These findings highlight the need to analyze not only species richness and abundance but also the potential for tick exchange among hosts and to correlate tick diversity with pathogen prevalence.

**Supplementary Information:**

The online version contains supplementary material available at 10.1007/s10493-026-01145-8.

## Introduction

Lagoon ecosystems are unique environments that harbor a wide diversity of flora and fauna. These systems are crucial for the health of both terrestrial and aquatic environments, providing essential environmental services such as water supply, climate change mitigation, and hydrological cycle regulation. Moreover, they generate high rates of primary and secondary productivity, which provides resources for both wildlife and humans (Gutiérrez et al. 2006; Castañeda and Contreras 2004). The Gulf of Mexico coastline hosts a significant portion of Mexico´s coastal lagoon systems; of the approximately 137 coastal lagoons nationally, about 45 occur along the Gulf of Mexico and Caribbean coast. Along the state of Veracruz coastline, 18 lagoon systems have been recognized (SEMARNAT 2003). Despite the valuable resources and environmental services, they provide these ecosystems have been significantly disturbed over the past 75 years. This degradation is driven by various anthropogenic activities for example urbanization, tourism, agriculture, and cattle ranching (González et al. 2017).

Cattle ranching represents a major economic activity in the state of Veracruz, both in terms of territorial extent—covering 50.6% of the state—and livestock inventory, with 2,723,963 head of cattle (INEGI 2022; Herrera 2005; Lizán et al. 2006). This cattle population has positioned Veracruz as the state with the highest cattle inventory in Mexico. This significant production context presents not only economic and competitiveness challenges but also serious sanitary and environmental issues, particularly relevant to the management and control of vector-borne diseases, especially those transmitted by ticks, whose life cycle and distribution can be influenced by extensive cattle production systems.

Tick infestations and the diseases they transmit are among the primary challenges impacting cattle production in grazing systems (García et al. 2016; Singh et al. 2022). In Mexico, ticks are distributed across six genera: *Dermacentor* (eleven species), *Haemaphysalis* (three species), *Rhipicephalus* (four species), *Ixodes* (28 species), *Robertsicus* (one species) and *Amblyomma* (25 species) (Guzmán-Cornejo et al. 2023). The genera *Ixodes* and *Amblyomma* exhibit the widest distribution in the country, with presence in 26 and 30 of Mexico’s 32 states, respectively (López-González et al. 2021; Guzmán-Cornejo et al. 2016, 2023). In the state of Veracruz, tick species from the Ixodidae family have been recorded; however, most reports have focused on species parasitizing livestock and/or companion animals (Aguilar-Domínguez et al. 2021; Rodríguez-Vivas et al. 2023). A significant knowledge gap remains regarding the diversity of ticks on co-occurring wild and domestic animals within lagoon ecosystems, as this specific host-tick relationship has not yet been investigated.

In this context, studying the diversity of ticks in these systems is important not only because of their role as vectors of various pathogens that pose risks to public and animal health, but also for developing more effective strategies for the control of these parasites and the diseases they transmit (Boulanger et al. 2019; OMS 2016). In the livestock sector in Mexico, ticks are responsible for the transmission of various infectious agents that affect cattle, such as *Babesia bovis* and *Anaplasma marginale*, which cause significant economic losses (Esteve-Gasent et al. 2020; Rojas-Martínez et al. 2021). Likewise, tick species carrying pathogens with zoonotic potential have been identified in wildlife, which increases the risk of transmission in ecosystems shared by humans and animals (Guglielmone et al. 2021).

In recent years, records of tick-borne diseases in humans have increased in several states of the Mexican Republic (for example, human borreliosis and / or rickettsiosis), including Veracruz (Frade-Ruiz 2018; Rodríguez-Muñoz et al. 2023; SINAVE 2025). Based on the above, the objective of this study was to assess the diversity and prevalence of ticks associated with cattle and wild animals coexisting in lagoon ecosystems of northern Veracruz, Mexico. This information is crucial for understanding vector-host ecological interactions in dynamic environments and for evaluating the potential risk of pathogen transmission.

## Materials and methods

### Study area

**Fig. 1:**
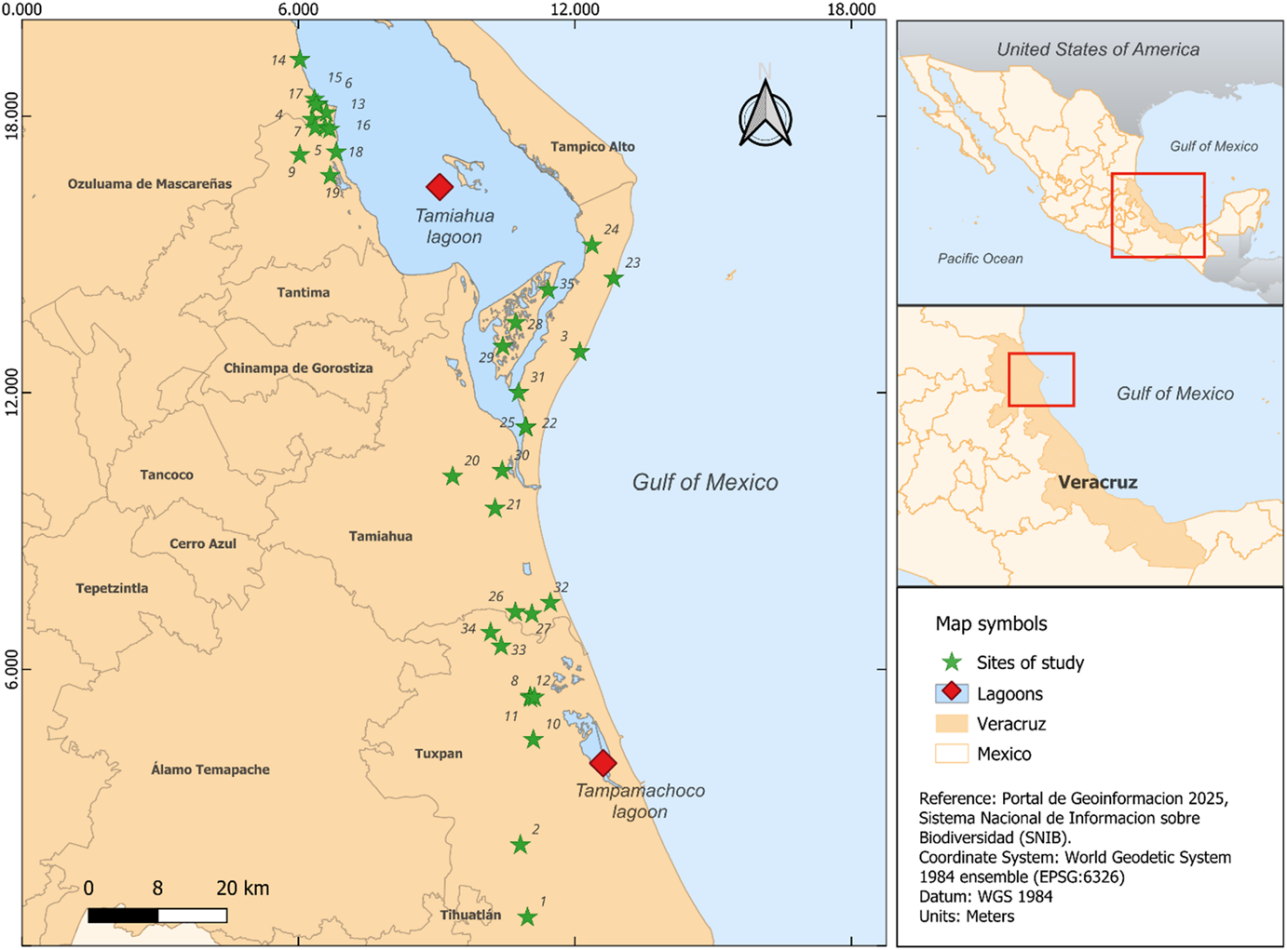
Location of the sampling sites in the northern region of Veracruz, Mexico. Prepared by García-Nolasco, DP (2025). Processed in QGIS 3.34.3 - Prizren (QGIS Development Team 2024)

The study was conducted from July 2022 to July 2023, encompassing both rainy and dry seasons, across 35 cattle production units (CPUs) located within the coastal lagoon ecosystem of the northern Veracruz, Mexico (Fig. 1). Each CPU was visited during five days (traps remained active for five consecutive nights), providing extended sampling effort despite the single visit-design. All CPUs were sited around two adjacent lagoon systems: Tamiahua and Tampamachoco Lagoons, which are separated by a short distance and embedded within the same tropical coastal plain. Tamiahua represents the largest lagoon system (≈ 88,000 ha), whereas Tampamachoco Lagoon is smaller (≈ 1,500 ha). Despite differences in size, both lagoon systems support comparable assemblages of domestic livestock and wildlife hosts, providing a suitable setting to evaluate tick-host associations at the wildlife-livestock interface. Due to their spatial continuity and similar environmental conditions, both lagoons were considered components of a single ecological study area (Fig. 1).

The region is characterized by a warm tropical climate, with mean annual temperatures generally exceeding 22° C and annual precipitation ranging from approximately 1,300 to 2,500 mm. Rainfall is seasonal, with a rainy period from June to October and a dry season from November to May. Mangrove communities, coastal dune vegetation, emergent aquatic plants, and patches of low deciduous and semi evergreen tropical forests, interspersed with agricultural and cattle grazing areas (Basáñez 2005; Morales and Paredes 2005) dominate vegetation surrounding the lagoon systems.

### Sample size calculation

A sample size of 385 animals was estimated using the WinEpi© (Working in Epidemiology) software, with a 50% expected prevalence, a 5% error margin, and a 95% confidence level. This calculation is valid for populations exceeding 1,000 individuals (Moreno-Manresa et al. 2018). Based on a total cattle population of 1,968,006 in northern Veracruz, this sample was proportionally inspected across the 35 CPUs (supplementary table). The selection criteria included cattle managed under extensive or semi-extensive grazing systems, those that were clinically healthy at the time of inspection, and those that had not been treated with acaricides for at least 14 days prior.

The study was approved by the Institutional Subcommittee for the Care and Use of Experimental Animals (SICUAE) of the Faculty of Veterinary Medicine and Zootechnics (FMVZ), Universidad Nacional Autónoma de México (UNAM) (Protocol number: SICUAE.DC-2022/2 − 1), as well as by the scientific collection permit granted by the Ministry of Environment and Natural Resources (SEMARNAT) (Permit No. SGPA/DGVS/03660/22). This last permit authorized the sampling of up to 50 rodents, 40 small mammals, and 30 medium-sized mammals and passerine birds.

### Capture of wild animals

Sampling sites were selected using a snowball scheme due to access difficulties. In each CPU, the following traps were deployed to capture various animal groups: 12 Tomahawk^®^ traps for medium-sized wildlife (González-Romero 2011), 60 Sherman^®^ traps for small mammals (mice and squirrels) (Cruz-Bazán et al. 2017), and five mist nets (6 and 10 m long, 1.5 cm mesh) for passerine birds (Correa 2015). Traps were baited with tuna, sardines, crab, corn, and squash for wild mammals; grapes and papaya for iguanas; and oatmeal with vanilla for squirrels and mice. Traps were arranged along linear transects and remained active for five consecutive nights. In general, traps were active from 17:00 h to 08:00 h the following morning for wild animals, and from 08:00 h to 13:00 h in the case of iguanas and squirrels. Mist nets were opened between 6:00 and 6:30 am and checked every 15 min until closed at 11:00 am (Cicuttin et al. 2019).

Wild animals were tranquilized and anesthetized according to species-specific protocols (Carpenter 2005; Arenas 2016; Flecknell 2009; Superina et al. 2014; Virbac n.d.), and birds were handled using physical restraint only. Subsequently, wildlife taxonomical determination was conducted using specialized identification keys for each group: mammals (Sánchez et al. 2015; González-Christen 2010), armadillos (Superina et al. 2014), rodents (Villalobos-Chaves et al. 2016), crocodiles (Sánchez et al. 2011), iguanas (O’Shea and Halliday 2001), and birds (González-Christen et al. 2016).

### Tick collection

For tick collection, all animals were inspected against the grain of their hair, from head to tail. For birds, only the head, neck, and wings were examined (Gammons and Salam 2002; Martínez-Sánchez et al. 2020). Once detected, ticks were carefully removed with forceps or manually, using a firm, upward motion to ensure complete extraction and prevent the detachment of mouthparts, which can cause infection or granuloma. The gnathosoma was preserved for taxonomic classification (Gammons and Salam 2002; Pulido-Villamarin et al. 2016). All collected ticks were placed in vials containing 96% ethanol for preservation and later analysis (Guzmán-Cornejo et al. 2020).

### Taxonomic identification of ticks

Adult ticks were identified based on taxonomic keys and descriptions of Guzmán-Cornejo et al. (2011) and Nava et al. (2014). Nymphal stages were identified using the morphological descriptions and identification keys of Martins et al. (2010). Larvae were identified to genus level based on morphological characters following the identification guide of Coley (2015), as species-level identification of immature stages is often limited by the lack of distinctive diagnosis characters.

Ticks collected from cattle were identified at the Parasitology Laboratory (Diagnostic Unit, Torreón del Molino ranch) of Universidad Veracruzana, using a Motic^®^ stereomicroscope; while ticks collected from wildlife (adults, nymphal and larval) were identified at the Acarology Laboratory, Faculty of Sciences, Universidad Nacional Autónoma de México, using a Nikon SMZ 645^®^ stereomicroscope.

### Statistical analysis

Tick prevalence, defined as the proportion of animals parasitized by at least one tick, was calculated as the number of infested hosts divided by the total number of examined animals and expressed as a percentage. It was calculated using the following formula: prevalence = (number of infested hosts / total hosts examined) × 100. Prevalence values and their corresponding 95% confidence intervals (95% CI) were estimated using descriptive statistics with the VassarStats online statistical tools (Lowry 2023).

Relative abundance (%) was estimated as the proportion of individuals of each tick species relative to the total number of ticks identified to species level. Calculations were based exclusively on adult and nymphal ticks and were expressed as percentages according to the formula: relative abundance (%) = (number of individuals of a given species / total number of adult and nymphal ticks) × 100. Larval specimens were excluded due to morphological limitations for species-level identification.

Alpha diversity analysis was performed using true diversity measures based on Jost’s framework (Jost 2006; Jost and González-Ortega 2012). Community composition was also assessed using rank-abundance curves, which account for species identity and complement diversity indices (Feinsinger 2003). Finally, host-tick interaction networks were constructed to visualize and quantify the relationships between host species and tick species. In these networks, each node represented either a host species or a tick species, and edges corresponded to observed infestations events. Edges were weighted according to the number of infested individuals per host species, allowing the networks reflect the relative strength of each interaction. Networks were analyzed based on connectance and interaction diversity using the *bipartite* package (Dormann et al. 2008) in R version 4.5 (R-Core Team 2025).

To differentiate between variations caused by species presence/absence and those due to changes in abundance, beta diversity analysis was used by comparing tick communities associated with cattle and wild animals. Two complementary indices were used: the Jaccard index to measure similarity based on species presence/absence and shared species proportion, and the Bray-Curtis index to quantify dissimilarity based on relative abundances between these host-associated communities (Schroeder and Jenkins 2018).

## Results

### Overall tick prevalence in cattle and wild animals

A total of 440 animals were examined, including 385 cattle and 55 wild animals (mammals, reptiles, and birds). Overall tick prevalence across the study area was 88.4% (389/440), 95% CI [84.9–91.1]. The prevalence was higher in cattle (93.7%) 95% CI [90.7–95.8] compared with wildlife (50.9%), 95% CI [37.2–64.4] (Table 1).

**Table 1:** Prevalence of ticks in cattle and wild animals in the study area

Host	Animals examined (*n*)	Infested animals	Prevalence (%)	95% CI
*Bos taurus*	385	361	93.7	90.7–95.8
Wild animals	55	28	50.9	37.2–64.4
Total	440	389	88.4	84.9–91.1

### Tick species composition and abundance

A comprehensive total of 7,026 ticks, representing various developmental stages, were collected across the lagoon ecosystem; this total includes 5,854 adults (83.3%), 984 nymphs (14%), and 188 larvae (2.7%). All specimens belonged to the family Ixodidae and were distributed among three genera (*Haemaphysalis*, *Rhipicephalus*, and *Amblyomma*). The most abundant species was *Rhipicephalus microplus* (71.1%), followed by *Amblyomma mixtum* (21.2%) (Table 2).

**Table 2:** Tick species composition by developmental stage and relative abundance

Genus	Species		Adults (*n*)	Nymphs (*n*)	Total (*n*)^1^	Relative abundance (%)^2^
*Rhipicephalus*		*Rhipicephalus microplus*	4,360	502	4,862	71.1
*Amblyomma*		*Amblyomma mixtum*	1,116	338	1,454	21.2
		*Amblyomma auricularium*	318	127	445	6.5
		*Amblyomma dissimile*	40	4	44	0.64
		*Amblyomma cf.* t*enellum*	1	8	9	0.13
		*Amblyomma ovale*	1	–	1	0.01
		*Amblyomma rotundatum*	1	–	1	0.01
*Haemaphysalis*		*Haemaphysalis leporispalustris*	17	5	22	0.32

### Tick–host associations

The allocation of tick species that parasitize both livestock and wild animals is presented in Table 3. The cattle population was infested by two distinct species of ticks, *R*. *microplus* and *A*. *mixtum*. Nevertheless, wild hosts were infested by a greater variety of ticks, particularly those belonging to the genus *Amblyomma.* Medium-sized mammals such as the opossum (*Didelphis virginiana*) and the raccoon (*Procyon lotor*) harbored the highest number of tick species, five and four species respectively (Table 3). Armadillos (*Dasypus novemcinctus*) and lagomorphs (*Sylvilagus floridanus*) hosted two tick species. In reptiles, *Amblyomma dissimile* was recorded on the green iguana (*Iguana iguana*), and *Amblyomma rotundatum* was found on the crocodile (*Crocodylus moreletii*). No adult or nymphal ticks were detected in the rodents and bird species examined.

**Table 3:** Tick species collected on both livestock and wild animal hosts

Order	Family	Host species	Hosts examined / infested (E/I)	Amblyomma mixtum	Rhipicephalus microplus	Amblyomma auricularium	Amblyomma dissimile	Amblyomma cf. tenellum	Amblyomma ovale	Amblyomma rotundatum	Haemaphysalis leporispalustris
Artiodactyla	Bovidae	*Bos taurus*	385 / 361	1129	4,852	–	–	–	–	–	–
Cingulata	Dasypodidae	*Dasypus novemcinctus*	5 / 5	164	–	382	–	–	–	–	–
Carnivora	Procyonidae	*Procyon lotor*	1 / 1	16	–	3	–	1	1	–	–
Lagomorpha	Leporidae	*Sylvilagus floridanus*	5 / 4	8	–	–	–	–	–	–	20
Didelphimorphia	Didelphidae	*Didelphis virginiana*	20/ 16	137	10	60	–	8	–	–	2
Rodentia	Sciuridae	*Sciurus aureogaster*	4 / 0	–	–	–	–	–	–	–	–
Rodentia	Muridae	*Mus musculus*	8 / 0	–	–	–	–	–	–	–	–
Squamata	Iguanidae	*Iguana iguana*	3 / 1	–	–	–	44	–	–	–	–
Passeriformes	Icteridae	*Psarocolius montezuma*	1 / 0	–	–	–	–	–	–	–	–
Passeriformes	Mimidae	*Mimus gilvus*	1 / 0	–	–	–	–	–	–	–	–
Passeriformes	Parulidae	*Geothlypis tricha*	2 / 0	–	–	–	–	–	–	–	–
Passeriformes	Thraupidae	*Thraupis episcopus*	1 / 0	–	–	–	–	–	–	–	–
Passeriformes	Tyrannidae	*Sayornis phoebe*	1 / 0	–	–	–	–	–	–	–	–
Passeriformes	Tyrannidae	*Pitangus sulphuratus*	1 / 0	–	–	–	–	–	–	–	–
Crocodylia	Crocodylidae	*Crocodylus moreletii*	1 / 1	–	–	–	–	–	–	1	–

### Diversity patterns of ticks

#### Alpha diversity of ticks by host group

**Fig. 2:**
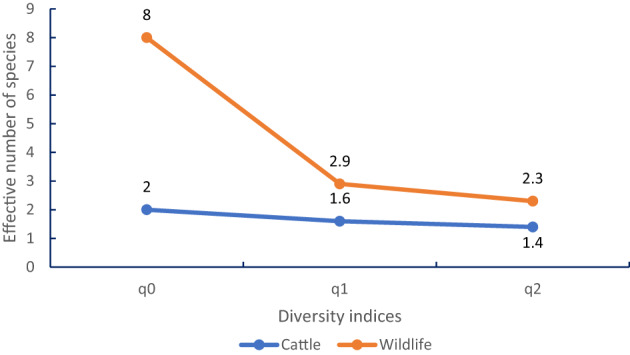
Diversity indices by host group, where q0 = observed species richness, q1 = Shannon´s index, q2 = Simpson´s index

The species richness (q0), the tick diversity represented by Shannon´s index (q1) and the inverse Simpson´s index, were higher in in wildlife hosts than in cattle (Fig. 2).

**Fig. 3:**
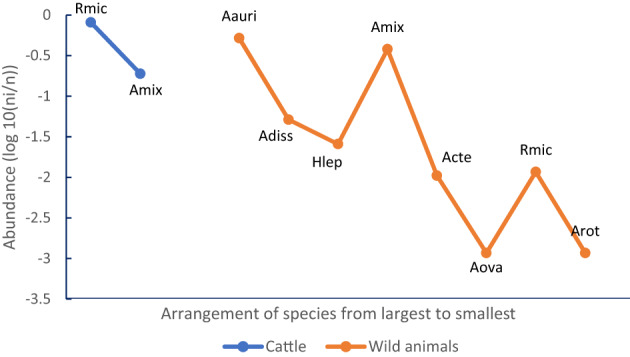
Rank-abundance curve for tick species by host group. Rmic: *Rhipicephalus microplus*, Amix: *Amblyomma mixtum*, Aaur: *Amblyomma auricularium*, Adis: *Amblyomma dissimile*, Hlep: *Haemaphysalis leporis palustris*; Acte: *Amblyomma* cf. *tenellum*, Aova: *Amblyomma ovale*, Arot: *Amblyomma rotundatum*. The X-axis represents the order of relative abundance (from highest to lowest), and the Y-axis represents the relative value of the proportion

The rank–abundance curve for tick in the host groups is shown in Fig. 3. In cattle, two species were observed, *R*. *microplus* as the most abundant species followed by *A*. *mixtum*. In wildlife, eight tick species were recorded (*A*. *auricularium*, *A. dissimile*, *H*. *leporispalustris*, *A. mixtum*, *A. cf. tenellum*, *A. ovale*, *R. microplus*, and *A. rotundatum*). These results indicated that wild animals exhibited greater tick species richness, with eight species recorded and a more even distribution among species (Fig. 3).

#### Diversity across the lagoon ecosystem

**Fig. 4:**
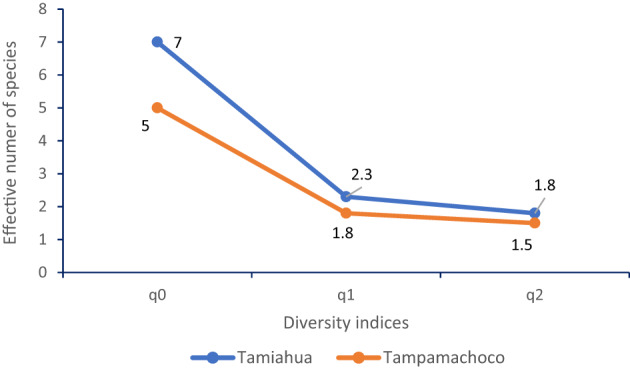
Diversity indices by lagoon, where q0 = observed species richness, q1 = Shannon´s index, q2 = Simpson´s index

The alpha diversity was examined descriptively by lagoon sub-area; the effective number of species was slightly higher in Tamiahua Lagoon than in Tampamachoco Lagoon (2.3 vs. 1.8). However, the values in the inverse Simpson index were low (Fig. 4).

**Fig. 5:**
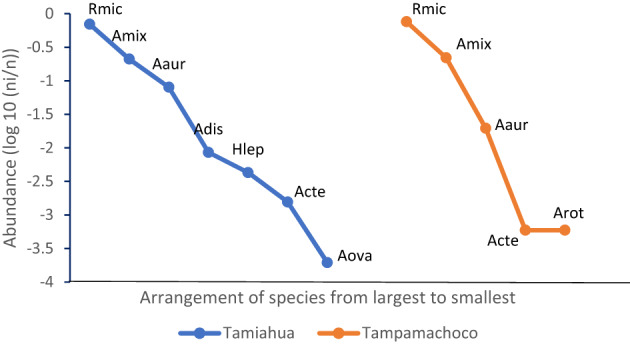
Rank-abundance curve for tick species in the two lagoons. Rmic: *Rhipicephalus microplus*, Amix: *Amblyomma mixtum*, Aaur: *Amblyomma auricularium*, Adis: *Amblyomma dissimile*, Hlep: *Haemaphysalis leporis palustris*; Acte: *Amblyomma* cf. *tenellum*, Aova: *Amblyomma ovale*, Arot: *Amblyomma rotundatum*. The X-axis represents the order of relative abundance (from highest to lowest), and the Y-axis represents the relative value of the proportion

The rank-abundance curve indicates that the structure of tick communities is very similar in the lagoons. This similarity is demonstrated by the consistent ordering of ectoparasite species, with *Rhipicephalus microplus* (Rmic) as the dominant species in both locations (Fig. 5).

### Ticks-host interaction in lagoon ecosystems

**Fig. 6:**
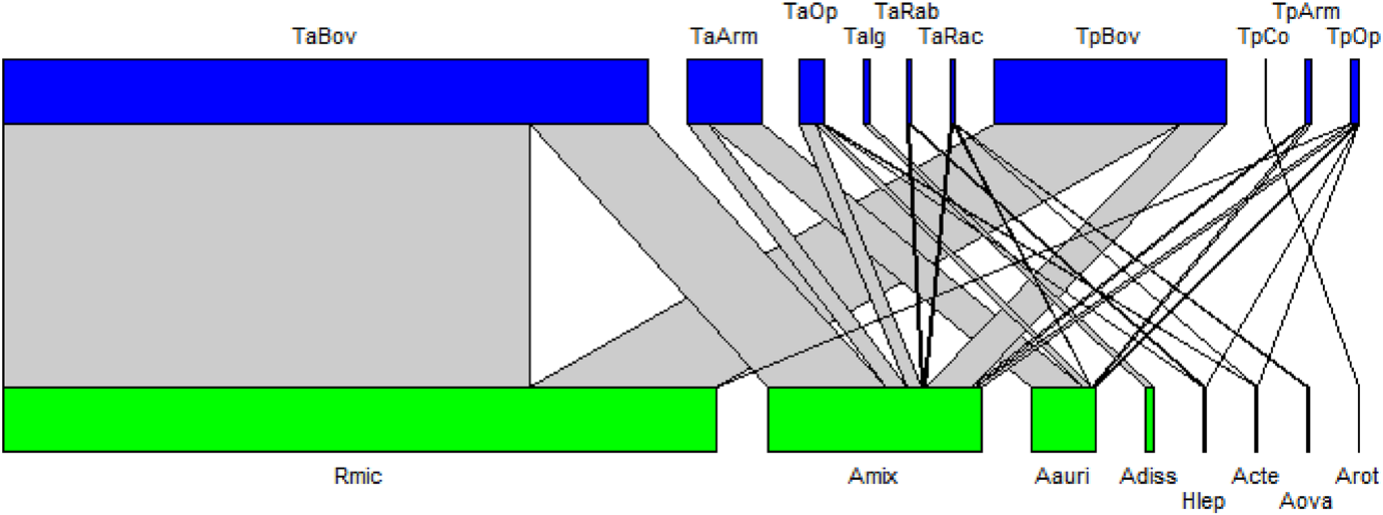
Interaction networks by tick species -host. The upper part (blue) shows the hosts. TaBov: Tamiahua cattle, TaArm: Tamiahua armadillos, TaOp: Tamiahua opossum, TaIg: Tamiahua Iguana, TaRab: Tamiahua rabbit, TaRac: Tamiahua raccoon, TpBov: Tampamachoco cattle, TpCoco: Tampamachoco crocodile, Tparm: Tampamachoco armadillo, TaOp: Tampamachoco opossum. The lower (green) section shows the tick species. Rmic: *Rhipicephalus microplus*, Amix: *Amblyomma mixtum*, Aaur: *Amblyomma auricularium*, Adis: *Amblyomma dissimile*, Hlep: *Haemaphysalis leporispalustris*; Acte:*Amblyomma* cf. *tenellum*, Aova: *Amblyomma ovale*, Arot: *Amblyomma rotundatum*

Analysis of the tick-host interaction networks revealed that *A*. *mixtum* exhibited the widest range of associations. These species were found predominantly on cattle, but also showed significant presence on wild animals, particularly *P. lotor*, *D*. *novemcinctus*,* D*. *virginiana* and *S*. *floridanus* (Fig. 6).

### Beta diversity

The Jaccard similarity index between cattle and wildlife was 0.25, indicating low similarity in tick species composition, as only two of the eight recorded species were shared. Moreover, the Bray-Curtis dissimilarity index registered a value of 0.90, indicating a considerable degree of dissimilarity in community structure due to marked variations in the relative abundance of species among the different host groups.

## Discussion

This study represents the first report on tick diversity associated with cattle and wild animals in lagoon ecosystems of northern Veracruz, Mexico. A total of eight tick species from the genera *Haemaphysalis*, *Rhipicephalus*, and *Amblyomma* were identified from vertebrate hosts, including reptiles, medium-sized mammals, and domestic cattle. Among wildlife, the genus *Amblyomma* was the most diverse. These ticks are characterized by their remarkable adaptability to a wide range of hosts, including mammals, birds, amphibians, and reptiles. This adaptability contributes to their wide distribution and ecological significance (Almazán et al. 2016; Palomo-Arjona et al. 2024; Busi et al. 2024). In the present study, *A. mixtum* was the most prevalent *Amblyomma* species of this genus recorded in both domestic and wild hosts. This species was found parasitizing five host species: *P*. *lotor*, *D*. *novemcinctus*, *D*. *virginiana*, *S*. *floridanus*, and *B*. *taurus*, indicating its occurrence across different host groups within the lagoon ecosystem. This finding is particularly relevant because *A*. *mixtum* was recorded parasitizing both wildlife and livestock hosts, suggesting its potential role in connecting tick populations across host communities.

Previous studies in Panama reported *A. mixtum* parasitism in raccoons (Bermúdez et al. 2015), indicating that this association is geographically widespread. Moreover, the generalist behavior of this tick species may be relevant for understanding potential pathogen circulation at the wildlife-cattle interface, although this was not directly evaluated in the present study. *Amblyomma mixtum* has also been identified as a potential vector for *Rickettsia rickettsii* (Gual-Gonzalez et al. 2024; Rivera-Páez et al. 2016) and a competent carrier of *R. amblyommatis* with isolations and detections reported across the Neotropics (Chaparro-Gutiérrez et al. 2023; Luna-Rojas et al. 2025). The abundance and host range represent key components in the ecological structure of tick communities and constitute a potential risk factor for public and animal health at the wildlife-domestic interface (Babayani and Makati 2021; Tsao et al. 2021). This supports the notion that some *Amblyomma* species exhibit high ecological plasticity, enabling them to exploit multiple hosts in anthropogenic landscapes and persist across diverse ecoregions (Aguilar-Domínguez et al. 2021; Gomes et al. 2024; Sánchez-Quirós and Barrantes 2024).

The detection of *A. mixtum* in wildlife underscores the generalist behavior of this tick species, a pattern that may be associated with the landscape transformation and habitat fragmentation characteristic of the anthropogenically modified lagoon ecosystems of northern Veracruz. This species is known for its ability to tolerate and persist in unstable or temporarily disturbed environments. Such resilience allows it to establish viable populations in mosaic landscapes, enabling coexistence with both wildlife and livestock and, consequently, favoring its high infestation levels (Forero-Becerra et al. 2022; Palomo-Arjona et al. 2024).

The presence of less common species, such as *A. ovale*, *A.* cf. *tenellum*, and *A. auricularium*, on raccoons (*P. lotor*) and armadillos (*D. novemcinctus*) is significant due to their potential role as pathogen vectors, despite their low abundance. For instance, *A. ovale* has been implicated in the transmission of *Rickettsia parkeri* strain Atlantic rainforest in wild carnivores (Aguirre et al. 2022; Lamattina et al. 2018). In this study, specimens morphologically consistent with *A*. cf. *tenellum* were recorded parasitizing wildlife hosts like opossums (*Didelphis virginiana*). Although the identification was conservative and therefore reported as cf., species of the genus *Amblyomma* are widely recognized as important vectors of several zoonotic pathogens, particularly bacteria belonging to the spotted fever group *Rickettsia*. In the Americas, members of the *A*. *cajennense* complex have been associated with pathogens such as *Rickettsia rickettsii* and other rickettsial agents of public health importance (Oliveira et al. 2010). Consequently, the occurrence of *A*. *cf. tenellum* on wildlife hosts may contribute to the maintenance of tick populations in natural environments and potentially facilitate pathogen circulation at the wildlife–livestock interface in coastal lagoon ecosystems (Nava et al. 2014). This finding suggests that these ticks may contribute to the maintenance of tick populations in natural environments and could potentially participate in pathogen circulation at the wildlife–livestock interface, where *Amblyomma* species act as bridge vectors contributing to the pathogen persistence in natural foci (Szabó et al. 2013). In addition, this finding aligns with the broader context of *Amblyomma* species as vectors of diverse pathogens (Benatti et al. 2020), underscoring the need for further studies to assess their vector competence within the (CPUs) of lagoon ecosystems.

In this study, the most abundant tick species in wild animals was *A*. *auricularium*, particularly in *D. novemcinctus*. This finding is consistent with previous reports describing *D. novemcinctus* as important hosts of this tick species (González-Álvarez et al. 2025). However, taxonomic uncertainty persists within *A*. *auricularium* across its geographic range. Specimens collected in Mexico belong to the group of non-ornamented ticks that have been sequenced, whereas ornamented forms have been reported from Brazil and Argentina. Resolving the taxonomic status of these populations will require additional sampling from Central America to clarify the identity and diversity of the species involved throughout its distribution (Guzmán-Cornejo et al. 2023).

Due to the co-occurrence of *A*. *auricularium* and *A*. *parvum* in armadillos, molecular confirmation is advisable, since adult morphological characters can overlap between both species. Recent surveys in Central American have explicitly combined morphological and molecular approaches to resolve ambiguous specimens (Badillo-Viloria et al. 2025), and previous studies have reported both species parasitizing the same hosts (Kluyber et al. 2016). This distinction is important because misidentification may lead to erroneous assignment of vector competence and inaccurate biodiversity assessments potentially resulting in under or overestimating of their true distribution. Moreover, *A*. *auricularium* and *A. parvum* belong to a natural group (Nava et al. 2008), defined as set of closely related species sharing a common evolutionary origin. This phylogenetic affinity suggests that both species derive from a relatively recent ancestral lineage, which explains their morphological similarity and the difficulty of distinguishing them based solely on external characters. Nevertheless, previous sub generic classifications do not fully reflect this relationship, reinforcing to need for a comprehensive taxonomic re-evaluation integrating both morphological and molecular evidence (Uribe et al. 2024). Clarifying these taxonomic relationships is essential for improving our understanding of the ecological dynamics and potential epidemiological roles of *Amblyomma* species at the wildlife-cattle production interface.

In this study, *H. leporispalustris* was identified in wild hosts *S*. *floridanus* and *D*. *virginiana*. This species has been reported in temperate and subtropical regions of the Americas (Guglielmone et al. 2021; Sánchez-Montes et al. 2020) and is commonly associated with lagomorphs, small mammals, and ground-dwelling birds that may act as reservoirs for several pathogens (Flores et al. 2023; Rodríguez-Vivas et al. 2023). The distribution of *H. leporispalustris* is largely influenced by the habitats of its principal hosts, particularly rabbits and other small vertebrates, which provide suitable ecological conditions for the maintenance of its populations. Although this species has been implicated in the transmission of pathogens associated with spotted fever group rickettsiae in some regions (Gabriele-Rivet et al. 2015), its epidemiological role in lagoon ecosystems of Mexico remains poorly understood. Therefore, further studies are required to evaluate its potential involvement in the maintenance and circulation of tick-borne pathogens in these ecosystems.

*R. microplus* was the dominant tick species found on cattle, which is consistent with reports from other tropical regions of Mexico, where it is considered the main ectoparasite in CPUs due to its high host specificity (Alonso-Díaz 2022). Although this species is strongly associated with cattle, it was also detected in a *D*. *virginiana*. Occasional infestations of *R*. *microplus* have been reported in non-domestic mammals and rarely on birds (Guglielmone et al. 2021), suggesting a limited but possible capacity to infest alternative hosts. Of the 7,026 ticks collected, 85.2% were obtained from cattle. This high abundance is likely related to factors such as the number of available hosts, their body size, their prolonged permanence within the same habitat, and the high density of cattle populations within the sampled CPU (Heylen et al. 2023).

The tick assemblage was strongly dominated by adult stages, which represented 83.3% of the collected specimens, whereas nymphs and larvae accounted for 14.0% and 2.7%, respectively. This pattern is commonly reported in studies where ticks are collected directly from hosts, as adult stages are larger and more easily detected during host examination (Omoregie et al. 2025). In the present study, nymphs were identified to species level, providing additional resolution for host–tick associations across developmental stages. Larvae were identified to genus level; however, given their low relative abundance, this limitation is unlikely to substantially influence the overall estimates of tick diversity. Therefore, the diversity patterns reported here provide a robust representation of the tick assemblage associated with the examined hosts.

### Diversity by host group

In this study, the greatest tick species richness was recorded in wild animals. However, only one livestock host species (*Bos taurus*) was examined, whereas wildlife hosts represented several taxonomic groups occupying diverse ecological niches, which may facilitate the maintenance of a greater diversity of tick species. This pattern is consistent with the diversity estimates (q0, q1, q2), where wildlife hosts showed greater effective diversity when species abundances were considered. In contrast, the tick community associated with cattle was strongly dominated by *Rhipicephalus microplus*, which reduced effective diversity despite the high number of ticks collected from this host. Similar patterns have been reported in other ecological studies, where wildlife hosts maintain a broader diversity of tick species, whereas domestic hosts are frequently dominated by a few highly adapted species (Guglielmone et al. 2014; Rodríguez-Vivas et al. 2016).

Moreover, several tick genera, particularly *Amblyomma* –and to a lesser extent *Ixodes* in other regions- show a strong affinity for wild hosts from different taxa, including small and medium-sized mammals, birds, and reptiles (Martínez-Sánchez et al. 2020; Guzmán-Cornejo et al. 2023). However, no *Ixodes* species were recorded in this study, which may reflect the specific environmental conditions and host composition of the lagoon ecosystem surveyed. These results highlight the ecological importance of *Amblyomma* species as dominant and versatile components of the tick community in northern Veracruz, particularly at the wildlife livestock interface.

Nevertheless, species diversity was greater in wildlife, supporting the hypothesis that wild hosts harbor a broader tick diversity and s may act as important reservoirs (Horak et al. 2022; Rodríguez-Durán et al. 2025; Rodríguez-Vivas et al. 2016). However, this pattern should be interpreted with caution, as only one domestic host species (*Bos taurus*) was evaluated in this study. Including additional domestic animals, such as dogs or horses, in future surveys would provide a more comprehensive comparison of tick diversity across host groups.

### Host–tick interactions in lagoon ecosystems

Analysis of host-tick interactions in these lagoon ecosystems revealed highly specific associations, such as *A. dissimile* on *Iguana iguana* and *A. rotundatum* on *Crocodylus moreletii*. This pattern, consistent with previously described parasitism in Neotropical reptiles (Guglielmone et al. 2021), suggests a possible host specialization and indicates that certain host-parasite interactions may persist despite the anthropogenic impacts in the lagoon ecosystem. However, given the very limited number of reptile hosts examined in this study (particularly a single *C*. *moreletii* individual) the representativeness of these findings remains uncertain. Additional surveys with larger sample sizes are needed to determine whether these interactions represent stable ecological associations.

Host–tick network analysis revealed notable differences in host connectivity. Among wildlife, *P. lotor* and *D. novemcinctus*, *S. floridanus* and *D. virginiana* functioned as central nodes, harboring a greater number of tick species compared to *I. iguana* and *C. moreletii*, which acted as peripheral nodes with more specific associations. *P*. *lotor* was parasitized by four different tick species in this study. Although this number is slightly lower than the six to eight species reported in other regions, it supports the general pattern of raccoons acting as multiparasitized hosts (Doi et al. 2018; Ito et al. 2024). Even if *Bos taurus* harbored fewer tick species than *P*. *lotor*, it also functioned as a central node, primarily due to its role as the main host for the dominant species *R. microplus* and *A. mixtum*. This finding suggests that cattle may also act as hosts connecting tick populations associated with wildlife and livestock, potentially facilitating the exchange of ticks at the wildlife- livestock interface.

Therefore, our network analysis reveals a host-parasite interaction pattern consistent with those reported in other Neotropical ecosystems. In the lagoon ecosystems of northern Veracruz, generalist mammals and domestic cattle act as central nodes in parasite dynamics, whereas reptiles and small mammals reinforce specialized interactions (Estrada-Peña et al. 2020). In this study, the tick diversity was estimated including adults and immature stages; thus, the observed host associations are aligned with the ecological roles described for different tick stages, where large mammals typically sustain adult infestations, but smaller vertebrates and birds may contribute to the maintenance of immature stages in natural environments.

### Sampling limitations

In this study, the very low capture success of birds and rodents likely contributed to the absence of adult ticks in these host groups. Bird sampling was restricted exclusively to passeriform species, excluding other avian taxa presents around study and that are more frequently reported as hosts of immature tick stages. Consequently, only eight birds were captured during the study. Likewise, despite deploying 60 live traps in grassland areas, only eight rodents were captured, indicating a very low capture success. These results may reflect habitat characteristics of open pasture landscapes, seasonal variation in small vertebrate activity, and low population densities associated with cattle production systems. As a result, the limited number of sampled individuals substantially reduced the probability of detecting tick infestations in birds and rodents. Therefore, the absence of ticks in these groups likely could reflects sampling limitations rather than a true absence of host–tick associations in the lagoon ecosystem, and this may have directly influence the observed diversity of ticks.

### Ecological implications

The lagoon ecosystems of northern Veracruz represent highly complex and dynamic environments. Their unique combination of ecological features -such as water bodies, riparian vegetation, mangroves, and agricultural areas-, facilitates frequent interactions between wild animals and cattle. However, the environmental heterogeneity of these systems also poses challenges for sampling, and it is possible that some host or tick species were underrepresented. The proximity of these ecosystems to coastal and mangrove habitats presents a potential risk, as it may facilitate the dispersal of ticks and associated pathogens to coastal fauna, including wild birds and mammals, thereby threatening their health. Although no adult ticks were collected from birds in this study, immature ticks were recovered exclusively as *Amblyomma* larval stages (*n* = 2). It is known that birds can host both adult and immature ticks, with larvae and nymphs being more frequently reported. In other Neotropical regions, previous studies have documented immature ticks and pathogens, such as bacteria of the genus *Rickettsia* in wild birds (Martínez-Sánchez et al. 2021).

## Conclusion

In conclusion, the study highlights a significant tick prevalence in both cattle and wild hosts within the lagoon ecosystems. Ticks were widely distributed among examined hosts, with a greater species diversity recorded in wild animals. Medium-sized mammals such as *D. virginiana* and *D. novemcinctus* were identified as hosts for the highest number of *Amblyomma* species, suggesting a complex ecological relationship that deserves further studies. The presence of *A*. *mixtum* in both host groups highlights the ecological connectivity between domestic and wild vertebrates within the lagoon ecosystem.

These findings emphasize the importance of the livestock–wildlife interface for understanding the maintenance and circulation of tick-borne pathogens in coastal ecosystems and highlight the need for future studies integrating tick diversity with pathogen surveillance.

## Supplementary Material

Below is the link to the electronic supplementary material.

Supplementary Material 1

## Data Availability

The authors declared that all the data are provided whitin this manuscript.
