# Supplementary material for: Diversity and prevalence of ticks associated to cattle and wild animals in lagoon ecosystems of northern Veracruz, México
Source: Exp Appl Acarol. 2026 May 27;97(1):3. doi: 10.1007/s10493-026-01145-8 (PMC13216078; doi:10.1007/s10493-026-01145-8)
Supplement: Supplementary file 1 — Supplementary Material 1 [file 10493_2026_1145_MOESM1_ESM.docx]

| **Supplementary table. Supplementary information on sampling site, coordinates, dates, hosts, tick species and stage. *Cattle production unit; **Date of animal capture** | | | | | | | |
| --- | --- | --- | --- | --- | --- | --- | --- |
| **CPU*** | **Latitude** | **Longitude** | **Date**** | **Host** | ***Tick*** | **Stage** | **Total** |
| Migueles | 20.837864 | -97.419884 | 07-11-22 | *Geothlypis trichas* | *-* | - | - |
|  |  |  | 05-11-22 | *Didelphis virginiana* | *-* | - | - |
|  |  |  | 04-11-22 | *Bos taurus* | *Rhipicephalus microplus* | Adult | 104 |
|  |  |  |  |  | *Rhipicephalus* | Nymph | 13 |
|  |  |  |  |  | *Amblyomma mixtum* | Adult | 54 |
| Villegas | 20.90852 | -97.429344 | 08-12-22 | *Geothlypis trichas* | *-* | - | - |
|  |  |  | 07-12-22 | *Didelphis virginiana* | *-* | - | - |
|  |  |  | 08-12-22 | *Didelphis virginiana* | *-* | - | - |
|  |  |  | 04-12-22 | *Mus musculus* | *-* | - | - |
|  |  |  | 03-12-22 | *Bos taurus* | *Rhipicephalus microplus* | Adult | 106 |
|  |  |  |  |  | *Rhipicephalus microplus* | Nymph | 28 |
|  |  |  |  |  | *Amblyomma mixtum* | Adult | 18 |
| Resumidero | 21.39306659 | -97.38153085 | 12-12-22 | *Thraupis episcopus* | *-* | - | - |
|  |  |  | 14-12-22 | *Geothlypis trichas* | *-* | - | - |
|  |  |  | 14-12-22 | *Mimus gilvus* | *-* | - | - |
|  |  |  | 11-12-22 | *Bos taurus* | *Rhipicephalus microplus* | Adult | 10 |
|  |  |  |  |  | *Amblyomma mixtum* | Adult | 75 |
| Paso del Mangle | 21.6133812 | -97.66866144 | 05-05-23 | *Mus musculus* | *Amblyomma* | Larvae | 1 |
|  | |  | 04-05-23 | *Mus musculus* | *Amblyomma* | Larvae | 1 |
|  | |  | 02-05-23 | *Didelphis virginiana* | *-* | - | - |
|  | |  | 30-04-23 | *Bos taurus* | *Rhipicephalus microplus* | Adult | 67 |
|  |  |  |  |  | *Rhipicephalus microplus* | Nymph | 2 |
| Peña | 21.60601638 | -97.65511395 | 12-05-23 | *Procyon lotor* | *Amblyomma Cf. tenellum* | Adult | 1 |
|  |  |  |  |  | *Amblyomma mixtum* | Adult | 1 |
|  |  |  |  |  | *Amblyomma mixtum* | Nymph | 15 |
|  |  |  |  |  | *Amblyomma ovale* | Adult | 1 |
|  |  |  |  |  | *Amblyomma auricularium* | Nymph | 3 |
|  |  |  |  |  | *Amblyomma* | Larvae | 62 |
|  |  |  | 09-05-23 | *Sylvilagus floridanus* | *Haemaphysalis leporispalustris* | Adult | 2 |
|  |  |  | 08-05-23 | *Bos taurus* | *Rhipicephalus microplus* | Adult | 99 |
|  |  |  |  |  | *Rhipicephalus microplus* | Nymph | 2 |
|  |  |  |  |  | *Amblyomma mixtum* | Adult | 5 |
| Las Flores | 21.62846546 | -97.66323833 | 25-04-23 | *Sciurus aureogaster* | *-* | - | - |
|  |  |  | 21-04-23 | *Bos taurus* | *Rhipicephalus microplus* | Adult | 138 |
|  |  |  |  |  | *Amblyomma mixtum* | Adult | 1 |
|  |  |  |  |  | *Rhipicephalus* | Larvae | 1 |
| Sandial | 21.60544624 | -97.66533082 | 17-05-23 | *Dasypus novemcinctus* | *Amblyomma mixtum* | Nymph | 119 |
|  |  |  |  |  | *Amblyomma auricularium* | Adult | 54 |
|  |  |  |  |  | *Amblyomma auricularium* | Nymph | 58 |
|  |  |  | 16-05-23 | *Didelphis virginiana* | *Haemaphysalis leporispalustris* | Nymph | 1 |
|  |  |  |  |  | *Haemaphysalis leporispalustris* | Nymph | 1 |
|  |  |  |  |  | *Amblyomma auricularium* | Nymph | 10 |
|  |  |  |  |  | *Amblyomma* | Larvae | 23 |
|  |  |  |  |  | *Amblyomma mixtum* | Nymph | 7 |
|  |  |  | 15-05-23 | *Sylvilagus floridanus* | *Amblyomma mixtum* | Nymph | 3 |
|  |  |  | 14-05-23 | *Bos taurus* | *Rhipicephalus microplus* | Adult | 99 |
|  |  |  |  |  | *Rhipicephalus microplus* | Nymph | 23 |
|  |  |  |  |  | *Amblyomma mixtum* | Adult | 2 |
| Cabaña | 21.05435411 | -97.42341925 | 08-07-23 | *Didelphis virginiana* | *Amblyomma mixtum* | Nymph | 22 |
|  |  |  |  |  | *Amblyomma Cf. tenellum* | Nymph | 1 |
|  |  |  |  |  | *Amblyomma* | Larvae | 21 |
|  |  |  |  |  | *Rhipicephalus microplus* | Adult | 10 |
|  |  |  | 06-07-23 | *Bos taurus* | *Rhipicephalus microplus* | Adult | 80 |
|  |  |  |  |  | *Rhipicephalus microplus* | Nymph | 42 |
|  |  |  |  |  | *Amblyomma mixtum* | Adult | 3 |
|  |  |  |  |  | *Amblyomma mixtum* | Nymph | 5 |
| Chomotla | 21.5783635 | -97.6806826 | 08-01-23 | *Sciurus aureogaster* | *-* | - | - |
|  |  |  | 06-01-23 | *Sayornis phoebe* | *-* | - | - |
|  |  |  | 05-01-23 | *Psarocolius montezuma* | *-* | - | - |
|  |  |  | 04-01-23 | *Bos taurus* | *Rhipicephalus microplus* | Adult | 208 |
|  |  |  |  |  | *Rhipicephalus microplus* | Nymph | 17 |
|  |  |  |  |  | *Amblyomma mixtum* | Adult | 197 |
| Don José | 21.012197 | -97.418844 | 17-07-23 | *Iguana iguana* | *-* | - | - |
|  |  |  | 14-07-23 | *Bos taurus* | *Rhipicephalus microplus* | Adult | 123 |
|  |  |  |  |  | *Amblyomma mixtum* | Adult | 140 |
|  |  |  |  |  | *Amblyomma mixtum* | Nymph | 2 |
| Tronconal | 21.052977 | -97.422948 | 05-08-22 | *Didelphis virginiana* | *Amblyomma auricularium* | Nymph | 4 |
|  |  |  | 31-07-22 | *Crocodylus moreletii* | *Amblyomma rotundatum* | Adult | 1 |
|  |  |  | 31-07-22 | *Bos taurus* | *Rhipicephalus microplus* | Adult | 85 |
|  |  |  | 31-07-22 |  | *Rhipicephalus microplus* | Nymph | 15 |
|  |  |  | 31-07-22 |  | *Amblyomma mixtum* | Adult | 42 |
| L3MA | 21.053586 | -97.418964 | 17-08-22 | *Sciurus aureogaster* | *-* | - | - |
|  |  |  | 15-08-22 | *Bos taurus* | *Rhipicephalus microplus* | Adult | 194 |
|  |  |  |  |  | *Rhipicephalus microplus* | Nymph | 2 |
|  |  |  |  |  | *Amblyomma mixtum* | Adult | 9 |
| Boca | 21.62024 | -97.654096 | 28-03-23 | *Didelphis virginiana* | *Amblyomma mixtum* | Nymph | 7 |
|  |  |  | 27-03-23 | *Didelphis virginiana* | *Amblyomma mixtum* | Nymph | 3 |
|  |  |  | 26-03-23 | *Bos taurus* | *Rhipicephalus microplus* | Adult | 200 |
|  |  |  |  |  | *Rhipicephalus microplus* | Nymph | 26 |
|  |  |  |  |  | *Amblyomma mixtum* | Adult | 1 |
| Herencia | 21.671548 | -97.683482 | 21-03-23 | *Didelphis virginiana* | *-* | - | - |
|  |  |  | 20-03-23 | *Bos taurus* | *Rhipicephalus microplus* | Adult | 113 |
|  |  |  |  |  | *Rhipicephalus microplus* | Nymph | 1 |
|  |  |  |  |  | *Amblyomma mixtum* | Adult | 34 |
| Salado | 21.633331 | -97.66695 | 18-03-23 | *Didelphis virginiana* | *Amblyomma mixtum* | Nymph | 30 |
|  |  |  |  |  | *Amblyomma auricularium* | Nymph | 11 |
|  |  |  |  |  | *Amblyomma* | Larvae | 1 |
|  |  |  | 17-03-23 | *Didelphis virginiana* | *Amblyomma auricularium* | Nymph | 24 |
|  |  |  |  |  | *Amblyomma mixtum* | Nymph | 2 |
|  |  |  |  |  | *Amblyomma* | Larvae | 1 |
|  |  |  |  |  | *Amblyomma Cf. tenellum* | Nymph | 7 |
|  |  |  | 14-05-23 | *Iguana iguana* | *Amblyomma dissimile* | Adult | 40 |
|  |  |  |  |  | *Amblyomma dissimile* | Nymph | 4 |
|  |  |  | 14-05-23 | *Bos taurus* | *Rhipicephalus microplus* | Adult | 165 |
|  |  |  |  |  | *Rhipicephalus microplus* | Nymph | 2 |
|  |  |  |  |  | *Amblyomma mixtum* | Adult | 1 |
|  |  |  |  |  | *Amblyomma mixtum* | Nymph | 1 |
| Rey | 21.604254 | -97.650128 | 04-04-23 | *Didelphis virginiana* | *Amblyomma mixtum* | Nymph | 10 |
|  |  |  | 02-04-23 | *Bos taurus* | *Rhipicephalus microplus* | Adult | 229 |
|  |  |  |  |  | *Rhipicephalus microplus* | Nymph | 18 |
|  |  |  |  |  | *Amblyomma mixtum* | Adult | 7 |
|  |  |  |  |  | *Amblyomma mixtum* | Nymph | 2 |
| Jardín | 21.628198 | -97.665101 | 08-03-23 | *Pitangus sulphuratus* | *Amblyomma* | Larvae | 2 |
|  |  |  | 11-03-23 | *Sylvilagus floridanus* | *Haemaphysalis leporispalustris* | Adult | 11 |
|  |  |  |  |  | *Haemaphysalis leporispalustris* | Nymph | 3 |
|  |  |  |  |  | *Haemaphysalis* | Larvae | 25 |
|  |  |  |  |  | *Amblyomma* | Larvae | 13 |
|  |  |  |  |  | *Amblyomma mixtum* | Nymph | 5 |
|  |  |  | 07-03-23 | *Bos taurus* | *Rhipicephalus microplus* | Adult | 171 |
|  |  |  |  |  | *Amblyomma mixtum* | Adult | 56 |
| Piedras | 21.582116 | -97.642481 | 19-04-23 | *Didelphis virginiana* | *Amblyomma mixtum* | Nymph | 7 |
|  |  |  |  |  | *Amblyomma auricularium* | Nymph | 11 |
|  |  |  | 16-04-23 | *Sciurus aureogaster* | *-* | - | - |
|  |  |  | 14-04-23 | *Bos taurus* | *Rhipicephalus microplus* | Adult | 171 |
|  |  |  |  |  | *Amblyomma mixtum* | Adult | 2 |
| Oro verde | 21.558834 | -97.648224 | 10-04-23 | *Didelphis virginiana* | *Amblyomma mixtum* | Nymph | 3 |
|  |  |  | 08-04-23 | *Bos taurus* | *Rhipicephalus microplus* | Adult | 172 |
|  |  |  |  |  | *Rhipicephalus microplus* | Nymph | 19 |
|  |  |  |  |  | *Amblyomma mixtum* | Adult | 95 |
|  |  |  |  |  | *Amblyomma mixtum* | Nymph | 2 |
| San Juan | 21.2675 | -97.510833 | 08-09-22 | *Didelphis virginiana* | *Amblyomma mixtum* | Nymph | 11 |
|  |  |  |  | *Dasypus novemcinctus* | *Amblyomma mixtum* | Adult | 1 |
|  |  |  |  |  | *Amblyomma auricularium* | Adult | 26 |
|  |  |  | 06-08-22 | *Bos taurus* | *Rhipicephalus microplus* | Adult | 97 |
|  |  |  |  |  | *Rhipicephalus microplus* | Nymph | 17 |
|  |  |  |  |  | *Amblyomma mixtum* | Adult | 17 |
| Parcela 9 | 21.237778 | -97.46556 | 02-07-23 | *Sylvilagus floridanus* | *-* | - | - |
|  |  |  | 01-07-23 | *Didelphis virginiana* | *Amblyomma mixtum* | Nymph | 32 |
|  |  |  | 30-06-23 | *Bos taurus* | *Rhipicephalus microplus* | Adult | 66 |
|  |  |  |  |  | *Rhipicephalus microplus* | Nymph | 6 |
|  |  |  |  |  | *Amblyomma mixtum* | Adult | 28 |
| Las chacas | 21.317796 | -97.435474 | 02-02-23 | *Bos taurus* | *Rhipicephalus microplus* | Adult | 131 |
|  |  |  |  |  | *Rhipicephalus microplus* | Nymph | 7 |
|  |  |  |  |  | *Amblyomma mixtum* | Adult | 33 |
| Miralobos | 21.4661261 | -97.3483015 | 23-05-23 | *Mus musculus* | *-* | - | - |
|  |  |  | 20-05-23 | *Bos taurus* | *Rhipicephalus microplus* | Adult | 119 |
|  |  |  |  |  | *Rhipicephalus microplus* | Nymph | 26 |
|  |  |  |  |  | *Amblyomma mixtum* | Adult | 2 |
| Providencia Ca. | 21.498056 | -97.371944 | 30-05-23 | *Mus musculus* | *-* | - | - |
|  | |  | 27-05-23 | *Bos taurus* | *Rhipicephalus microplus* | Adult | 127 |
|  | |  |  |  | *Rhipicephalus microplus* | Nymph | 56 |
|  | |  |  |  | *Rhipicephalus* | Larvae | 8 |
|  | |  |  |  | *Amblyomma mixtum* | Adult | 2 |
| Chino | 21.317889 | -97.436306 | 09-02-23 | *Bos taurus* | *Rhipicephalus microplus* | Adult | 101 |
|  |  |  |  |  | *Rhipicephalus microplus* | Nymph | 30 |
|  |  |  |  |  | *Amblyomma mixtum* | Adult | 2 |
| Ritchie | 21.136739 | -97.441328 | 25-07-23 | *Dasypus novemcinctus* | *Amblyomma mixtum* | Adult | 5 |
|  |  |  |  |  | *Amblyomma mixtum* | Nymph | 5 |
|  |  |  |  |  | *Amblyomma* | Larvae | 1 |
|  |  |  |  |  | *Amblyomma auricularium* | Adult | 58 |
|  |  |  | 20-07-23 | *Bos taurus* | *Rhipicephalus microplus* | Adult | 140 |
|  |  |  |  |  | *Rhipicephalus microplus* | Nymph | 26 |
|  |  |  |  |  | *Amblyomma mixtum* | Adult | 6 |
| Ciénega | 21.135302 | -97.423984 | 30-07-23 | *Dasypus novemcinctus* | *Amblyomma auricularium* | Adult | 165 |
|  |  |  |  |  | *Amblyomma auricularium* | Nymph | 2 |
|  |  |  |  |  | *Amblyomma mixtum* | Adult | 6 |
|  |  |  |  |  | *Amblyomma mixtum* | Nymph | 1 |
|  |  |  |  |  | *Amblyomma* | Larvae | 5 |
|  |  |  | 26-07-23 | *Bos taurus* | *Rhipicephalus microplus* | Adult | 19 |
|  |  |  |  |  | *Rhipicephalus microplus* | Nymph | 19 |
|  |  |  |  |  | *Amblyomma mixtum* | Adult | 9 |
| Pelo | 21.420191 | -97.449404 | 12-06-23 | *Bos taurus* | *Rhipicephalus microplus* | Adult | 72 |
|  |  |  |  |  | *Amblyomma mixtum* | Adult | 10 |
| El potro | 21.396312 | -97.462265 | 19-06-23 | *Bos taurus* | *Rhipicephalus microplus* | Adult | 220 |
|  |  |  |  |  | *Rhipicephalus microplus* | Nymph | 15 |
|  |  |  |  |  | *Rhipicephalus* | Larvae | 2 |
|  |  |  |  |  | *Amblyomma mixtum* | Adult | 9 |
| Ceja | 21.274483 | -97.459225 | 10-06-23 | *Sylvilagus floridanus* | *Haemaphysalis leporispalustris* | Adult | 4 |
|  |  |  |  |  | *Amblyomma* | Larvae | 3 |
|  |  |  |  |  | *Haemaphysalis* | Larvae | 2 |
|  |  |  | 06-06-23 | *Bos taurus* | *Rhipicephalus microplus* | Adult | 14 |
|  |  |  |  |  | *Rhipicephalus microplus* | Nymph | 15 |
|  |  |  |  |  | *Amblyomma mixtum* | Adult | 145 |
|  |  |  |  |  | *Amblyomma mixtum* | Nymph | 3 |
| Josefa | 21.351404 | -97.444432 | 15-02-23 | *Bos taurus* | *Rhipicephalus microplus* | Adult | 169 |
|  |  |  |  |  | *Rhipicephalus microplus* | Nymph | 23 |
|  |  |  |  |  | *Amblyomma mixtum* | Adult | 54 |
|  |  |  |  |  | *Amblyomma mixtum* | Nymph | 8 |
| Encanto | 21.146893 | -97.404997 | 30-12-22 | *Didelphis virginiana* | *Amblyomma mixtum* | Nymph | 3 |
|  |  |  | 28-12-22 | *Mus musculus* | *-* | - | - |
|  |  |  | 27-12-22 | *Iguana iguana* | *-* | - | - |
|  |  |  | 26-12-22 | *Bos taurus* | *Rhipicephalus microplus* | Adult | 171 |
|  |  |  |  |  | *Rhipicephalus microplus* | Nymph | 3 |
| Insunza | 21.10293 | -97.455143 | 25-07-22 | *Didelphis virginiana* | *Amblyomma* | Larvae | 16 |
|  |  |  | 20-07-22 | *Bos taurus* | *Rhipicephalus microplus* | Adult | 124 |
|  |  |  |  |  | *Rhipicephalus microplus* | Nymph | 34 |
|  |  |  |  |  | *Amblyomma mixtum* | Adult | 9 |
|  |  |  |  |  | *Amblyomma mixtum* | Nymph | 4 |
| Cedros | 21.116061 | -97.466579 | 19-10-22 | *Dasypus novemcinctus* | *Amblyomma mixtum* | Adult | 1 |
|  |  |  |  |  | *Amblyomma mixtum* | Nymph | 26 |
|  |  |  |  |  | *Amblyomma auricularium* | Adult | 15 |
|  |  |  |  |  | *Amblyomma auricularium* | Nymph | 4 |
|  |  |  | 15-10-22 | *Bos taurus* | *Rhipicephalus microplus* | Adult | 142 |
|  |  |  |  |  | *Rhipicephalus microplus* | Nymph | 7 |
|  |  |  |  |  | *Amblyomma mixtum* | Adult | 27 |
| Porvenir Id. | 21.45288 | -97.41658 | 24-06-23 | *Bos taurus* | *Rhipicephalus microplus* | Adult | 104 |
|  |  |  |  |  | *Rhipicephalus microplus* | Nymph | 8 |
|  |  |  |  |  | *Amblyomma mixtum* | Adult | 7 |
